# Clinical efficacy of bladder function training combined with pelvic floor biofeedback electrical stimulation on neurogenic bladder and its impact on urodynamics

**DOI:** 10.12669/pjms.41.1.9655

**Published:** 2025-01

**Authors:** Jiawei Gu, Jun Zang

**Affiliations:** 1 Jiawei Gu Department of Urology, Beijing Shijingshan Hospital, Beijing 100040, Beijing, China; 2 Jun Zang Neurological Rehabilitation Center, Beijing Rehabilitation Hospital of Capital Medical University, Beijing 100000, Beijing, China

**Keywords:** Bladder Function Training, Pelvic Floor Biofeedback Electrical Stimulation, Neurogenic Bladder, Urodynamics, Treatment

## Abstract

**Objective:**

To evaluate the clinical efficacy of bladder function training combined with pelvic floor biofeedback electrical stimulation in the treatment of neurogenic bladder and its impact on urodynamics.

**Methods:**

This was a clinical comparative study. A total of 120 patients with neurogenic bladder after spinal cord injury admitted to Beijing Rehabilitation Hospital of Capital Medical University and Beijing Shijingshan Hospital from January 2023 to December 2023 were randomly divided into two groups (n= 60/group). The study group treated with bladder function training combined with pelvic floor biofeedback electrical stimulation and the control group receiving conventional treatment and bladder function training. The differences in the clinical efficacy, voiding diary, voiding symptom score, urodynamics parameters, etc. were between the two groups were compared and analyzed.

**Results:**

The difference of the efficacy in the study and control groups was statistically significant(p=0.02). After treatment, the study group showed significant improvement compared to the control group in terms of daily mean voiding frequency, daily mean single urine volume, daily maximum single urine volume, and catheterization frequency(P=0.00). Meanwhile, the study group also saw significantly lower residual urine volume (RUV) than the control group, with statistically significant differences(p=0.00), while MBC, Qmax, and BP were significantly higher in the study group than in the control group, with statistically significant differences(p=0.00).

**Conclusion:**

The application of bladder function training combined with pelvic floor biofeedback electrical stimulation is significantly effective for treating neurogenic bladder, which can significantly improve the storage and voiding functions of patients, alleviate lower urinary tract symptoms, and improve patients’ quality of life.

## INTRODUCTION

Also known as neurogenic lower urinary tract dysfunction, neurogenic bladder(NB) is a common complication after central nervous system injury and is accompanied by bladder dysfunction in about 10% of patients with multiple sclerosis, 30-70% of those with Parkinson’s disease, about 37-48% of patients with stroke, and a considerable number of patients with spinal cord injury.^1^ Most patients experience varying degrees of reduced bladder compliance, increased intravesical pressure, etc., which can lead to risks such as urinary tract infection and renal failure, ultimately increasing patients’ suffering and severely impacting their quality of life.^2^,^3^ At present, the clinical treatment methods for neurogenic bladder mainly include drug therapy, rehabilitation therapy, and neuromodulation techniques, the efficacy of which is subject to some controversy. Meanwhile, bladder function training aims to increase bladder capacity and improve bladder function through methods such as scheduled drinking to prolong the urination interval, which demonstrates certain clinical value in the recovery of neurogenic bladder.^4^

However, it has not yet achieved the desired therapeutic effect. In contrast, electrical stimulation involves the generation of stimulating signals at specific sites through electric currents, transmitting excitatory signals to the pelvic nerves, and producing signal feedback in the spinal cord to improve bladder function. At the same time, pelvic floor biofeedback electrical stimulation (PES) is a novel technique introduced in recent years, which works by instructing patients through training using electrical stimulation, visual signals, and voice prompts. This combined approach enhances the participation and motivation of patients with pelvic floor muscle training and instructs them to exercise in a controllable way, thereby effectively improving their bladder function.^5^ This study was primarily designed to explore the clinical efficacy of bladder function training combined with pelvic floor biofeedback electrical stimulation in the treatment of neurogenic bladder and its impact on urodynamics.

## METHODS

This was a clinical comparative study. A total of 120 patients with neurogenic bladder after spinal cord injury admitted to Beijing Rehabilitation Hospital of Capital Medical University and Beijing Shijingshan Hospital from January 2023 to December 2023 were randomly divided into the study group and the control group (n= 60) according to different treatment methods. Patient data comes from the electronic medical record system in our hospital. According to the data of each indicator in the pre-survey, the sample size is estimated by 95% confidence interval, and the largest one is the sample size of the study. The sample size required for each group was ≥60 cases on the basis of Fisher exact probability.

### Ethical Approval:

The study was approved by the Institutional Ethics Committee of Beijing Shijingshan Hospital (No.: sjsky2023-12; date: December 01, 2023), and written informed consent was obtained from all participants.

### Inclusion criteria:

- Patients who met the diagnostic criteria for neurogenic bladder^6^ and had symptoms of difficulty urinating and urinary retention;
- Patients who were conscious and able to cooperate with training;
- Patients with ASIA neurological function grade B~D;
- Patients with stable condition and without combined injuries;
- Age 30~70 years old;
- Patients with complete clinical data;
- Patients and their families agreed to participate in the study and signed informed consent.

### Exclusion criteria:

- Patients with severe heart, liver, or other organ diseases;
- Patients with a history of kidney disease;
- Patients with bladder muscle injury;
- Patients with severe urinary system infections that cannot be effectively controlled;
- Patients with a history of other diseases affecting urination function such as urethral stricture, chronic cystitis, prostate enlargement, bladder neck contracture, etc.;
- Patients with contraindications to pelvic floor biofeedback electrical stimulation;
- Patients without complete clinical data;
- Patients with mental or neurological disorders who cannot satisfactorily cooperate with the study.

The study group was treated with bladder function training combined with pelvic floor biofeedback electrical stimulation, while the control group received conventional treatment and bladder function training. The average age of the study and control groups was 56.72±12.01 years (35- 68 years old) and 56.83±12.66 years (34 - 70 years old), respectively, with no significant differences in general patient data between the two groups (p>0.05), indicating inter-group comparability (Table-I).

**Table-I:** Comparison of Patient Profile between the two Groups(*χ̅*±*S*) n=60.

Indicator	Study Group	Control Group	t/χ^2^	p
Age(years)	56.72±12.01	56.83±12.66	0.05	0.96
Course of disease(years)	3.27±0.83	3.46±0.75	1.32	0.19
ASIA Grade				
Grade B(%)	32(53%)	35(58%)	0.30	0.58
Grade C(%)	20(33%)	15(25%)	1.01	0.32
Grade D(%)	8(14%)	10(17%)	0.26	0.61
Disease Type				
Upper motor neuropathy (%)	11(18%)	13(22%)	0.21	0.65
Spinal cord lesion (%)	16(27%)	15(25%)	0.40	0.52
Lower motor neuropathy (%)	33(55%)	32(53%)	0.03	0.86

p>0.05.

### Methods:

The control group received basic treatment and conventional bladder function training, with the former including nutritional nerve support, anti-inflammatory therapy, and treatment of primary diseases. Bladder function training:

### Water intake:

A water intake plan was formulated, with patients drinking 200 ml of water every two h after waking up, deducting water content from food, and aiming for an average daily water intake of less than 2 L

### Triggering urination reflex:

Finding the trigger point for the urination reflex through tapping, pulling, and pinching to help patients establish spontaneous urination reflex, 10 times/day.

During urination, patients were assisted in urination by squeezing their abdomen with a fist, three times/day for two min/time.

### Levator ani muscle training:

Contraction exercises of the levator ani muscle were performed without contracting the abdominal, gluteal, or leg muscles, 50 times per day, with contraction time prolonged per training progress.

### Delayed urination training:

Patients who felt the urge to urinate were instructed to delay urination for 10 min before urination, three times/day.

The study group received pelvic floor biofeedback electrical stimulation (PES) in addition to the treatment used in the control group, with the application of a PES therapy device. Specifically, patients were placed in a lateral or supine position, with the conductive gel applied to the therapeutic rod and inserted into the rectum (unmarried females and males) or vagina (married females) to a depth of 5-7 cm for pelvic floor biofeedback electrical stimulation. Parameters: Frequency 20-35 Hz, pulse width 200-220 m, continuous stimulation for one minute, followed by a 30 seconds interval, then another one minute stimulation, once a day for 30 minutes. The treatment intensity was adjusted based on individual patient tolerance to ensure muscle contraction or pulsation without pain. Meanwhile, patients were also instructed to perform anal sphincter training. The therapist instructed the patients to contract the anus to the maximum extent and collected the maximum sphincter surface EMG signal to use as the baseline, followed by asking them to contract the anus again and encouraging them to try to surpass that baseline for five seconds before relaxing. During each treatment session, the therapist instructed the patients to contract and relax the pelvic floor muscles based on the biofeedback curves obtained, adjusting the intensity of pelvic floor biofeedback electrical stimulation based on the exercise volume of patients, three times/day for 10-20 minutes/time. Ultimately, both groups were treated for four weeks, and followed up for three months.

### Outcome Indicators:

### Clinical efficacy assessment:

Significantly effective: Urination under conscious control, presence of urination reflex, residual urine volume <50 ml; Effective: Urination under conscious control, presence of urination reflex, residual urine volume = 50-150 ml; Ineffective: Inability to establish urination reflex, uncontrolled urination, residual urine volume < 400 ml. Overall response rate = (significantly effective + effective) ÷ total number × 100%^7^;

### Voiding diary:

The daily average urination frequency, daily average single urine volume, daily maximum single urine volume, and catheterization frequency were recorded;

### Urodynamic parameters:

The residual urine volume (RUV), maximum bladder capacity (MBC), maximum flow rate (Qmax), and bladder pressure (BP) was detected using a urodynamic analyzer;

### Voiding symptom score:

Urinary symptom distress score (USDS)^8^ and lower urinary tract symptoms (LUTS)^9^ were utilized to assess the voiding symptoms of patients. Urinary incontinence is defined as the uncontrolled outflow of urine from the bladder. The USDS score ranges from delighted to very distressed, i.e., 0-6 points, with a total score range of six points, while the LUTS scale contains seven questions, scored from 0-5 points, with a total score range of 0-35 points, in which a higher score indicates more severe symptoms;

### Quality of Life:

A comparative analysis of the improvement in quality of life for both groups pre- and post-treatment was conducted using the Quality of Life Assessment (QOL) and SF-36 Scale.^10^

### Statistical Analysis:

SPSS 20.0 software was used for the statistical analysis of all data, and measurement data were expressed as (±S). Inter-group data analysis was performed using two independent samples t-tests. In the study group, a paired t-test was utilized for comparative analysis of each indicator pre- and post-treatment, and a χ^2^ test was used for comparison of rates. P< 0.05 was considered statistically significant.

## RESULTS

The comparison of the therapeutic effect between the two groups showed a response rate of 82% and 62% in the study and control groups, respectively, and the former exhibited a significantly higher rate than the latter, with statistically significant differences (p= 0.02), (Table-II).

**Table-II:** Comparative analysis of therapeutic effect between the two groups (*χ̅*±*S*)n=60.

Group	Significantly effective	Effective	Ineffective	Overall response rate*
Study	25	24	11	49(82%)
Control	19	18	23	37(62%)
*χ* ^2^				5.91
*p*				0.02

* p<0.05.

No significant difference was observed in daily average urination frequency, daily average single urine volume, daily maximum single urine volume, and catheterization frequency between the two groups pre-treatment(p> 0.05), and these indicators in the study group were significantly improved compared with the control group post-treatment, and the differences were significant (P = 0.00) (Table-III).

**Table-III:** Comparative analysis of voiding diary content between the two groups(*χ̅*±*S*)n=60.

Indicator	Group	Study	Control	t	p
Daily average urination frequency (time)	Pre-treatment	2.38±1.02	2.33±1.10	0.26	0.80
	Post-treatment*	5.48±0.53	4.20±0.62	12.15	0.00
Daily average single urine volume (ml)	Pre-treatment	117.83±11.07	117.05±10.45	0.55	0.58
	Post-treatment*	243.50±30.88	215.96±27.41	5.38	0.00
Daily maximum single urine volume(ml)	Pre-treatment	60.87±16.21	61.08±17.34	0.07	0.94
	Post-treatment*	120.65±15.10	87.59±12.03	12.90	0.00
Catheterization frequency (time)	Pre-treatment	5.08±1.13	5.12±1.08	0,20	0.84
	Post-treatment*	3.32±1.53	3.98±1.50	2.73	0.00

* p<0.05.

No significant difference was found in RUV, MBC, Qmax, and BP between the two groups pre-treatment (p> 0.05), and RUV in the study group was significantly lower than that in the control group post-treatment, with statistically significant differences (p= 0.00). Meanwhile, MBC, Qmax, and BP in the study group were significantly higher than those in the control group, and the differences were statistically significant (p= 0.00), (Table-IV).

**Table-IV:** Comparative analysis of urodynamic parameters(*χ̅*±*S*) n=60.

Indicator	Group	Study	Control	t	p
RUV(ml)	Pre-treatment	270.20±32.61	278.39±34.47	1.13	0.18
	Post-treatment*	76.64±12.07	117.52±24.63	11.84	0.00
MBC(ml)	Pre-treatment	153.07±15.08	157.42±16.42	1.41	0.16
	Post-treatment*	382.29±35.78	296.43±40.46	12.53	0.00
Qmax(ml/s)	Pre-treatment	11.16±3.73	11.04±3.38	2.83	0.17
	Post-treatment*	16.70±3.18	12.70±3.05	7.30	0.00
BP(cmH_2_O)	Pre-treatment	12.08±2.35	12.32±2.67	0.52	0.60
	Post-treatment*	25.40±2.48	22.16±2.30	7.42	0.00

* p<0.05.

There were no significant differences in USDS and LUTS scores between the two groups pre-treatment (p > 0.05), and these scores in the study group were significantly lower than those in the control group post-treatment, with statistically significant differences (p= 0.00), (Table-V).

**Table-V:** Comparative analysis of voiding symptom scores between the two groups(*χ̅*±*S*) n=60

Indicator	Group	Study	Control	t	p
USDS Score	Pre-treatment	5.03±0.78	4.97±0.62	0.47	0.64
	Post-treatment*	2.93±0.14	3.18±0.20	7.93	0.00
LUTS Score	Pre-treatment	30.32±3.08	30.49±3.11	0.30	0.76
	Post-treatment*	16.43±2.45	18.30±2.24	4.36	0.00

* p<0.05.

No significant differences were observed in pre-treatment QOL and SF-36 scores between the two groups (p > 0.05). However, the post-treatment QOL score in the study group was significantly lower than that in the control group, and the post-treatment SF-36 score was significantly higher than that in the control group, with statistically significant differences (p= 0.00), (Table-VI).

**Table-VI:** Comparison of pre- and post-treatment QOL scores between the two groups(*χ̅*±*S*) n=60.

Indicator	Group	Study	Control	t	p
QOL Score	Pre-treatment	5.12±1.08	5.09±1.16	0.14	0.88
	Post-treatment*	2.09±0.31	3.32±0.28	7.93	0.00
SF-36 Score	Pre-treatment	46.06±7.48	46.15±7.62	0.36	0.62
	Post-treatment*	62.15±9.17	55.31±8.72	5.86	0.00

* p<0.05.

## DISCUSSION

This study indicates a response rate of 82% and 62% in the study and control groups, respectively, with statistically significant differences(p=0.02), which confirms the significant clinical efficacy of bladder function training combined with pelvic floor biofeedback electrical stimulation (PES) in treating neurogenic bladder. On the one hand, as a non-invasive and simple treatment method, PES involves electrically stimulating the pelvic floor muscles with a therapeutic electrode inserted into the rectum or vagina to prompt their contraction, followed by instructing patients to control the contraction and relaxation of the pelvic floor muscles, thereby improving their voiding function. On the other hand, PES belongs to low-frequency electrical stimulation therapy, which can promote the recovery of bladder sensory function, allow patients to accurately perceive bladder capacity, determine whether they need to empty their bladder, and instruct patients to urinate within a safe capacity period in time according to urodynamics, thus protecting their upper urinary tract. Leonardo et al.^11^ have concluded that pelvic floor muscle biofeedback treatment can effectively promote bladder function recovery and spontaneous urination in NB patients after SCI, demonstrating significant effects.

With the advantages of fewer side effects and less trauma compared to drug and surgical treatments, bladder function training involves regulating bladder capacity through timed water intake, allowing the bladder to contract and expand according to different capacities, which helps patients establish spontaneous urination reflex by stimulating the trigger points of the urination reflex, ultimately preventing urinary reflux-induced complications due to high bladder pressure.^12^,^13^ Through continuous training and reinforcement, patients gradually gain control over urination, and this simple and effective training method is widely applied in the treatment of neurogenic bladder. However, for some severely affected patients with poor bladder muscle function, bladder function training alone may not be effective, and therefore may not lead to significant rehabilitation effects. In view of this, stimulation therapy combined with bladder function training may be more effective in the rehabilitation of neurogenic bladder in patients with spinal cord injury. Specifically, stimulation therapy involves stimulating the nerves and muscle tissues around the patient’s bladder with low-frequency electrical currents, inducing contractions of the urethral sphincter and bladder detrusor muscle^14^. Moreover, studies suggest that functional exercise combined with stimulation therapy may be more effective than monotherapy.^15^,^16^

This study proved that bladder function training combined with PES could better improve the voiding and urodynamic conditions of patients with neurogenic bladder after spinal cord injury compared to bladder function training alone. This is because PES can stimulate the sympathetic nerves, inhibit the parasympathetic nerves, reduce bladder contraction ability, and enlarge bladder capacity.^17^ Meanwhile, it can also enhance the neuromuscular excitability of the pelvic floor muscles by stimulating the rectum or vagina, thereby enhancing the function of the pelvic floor muscle and urethral sphincter. Since electrical stimulation can effectively enhance bladder emptying capacity, PEF can improve bladder function in patients.^18^ Moreover, some studies have also indicated that through stimulation of impulses ascending to the thoracolumbar region and sympathetic neurons, α-adrenergic receptors can promote constriction of the bladder neck and proximal urethra to enhance urethral closure function and regulate lower urinary tract function, thus improving the bladder function of patients by enhancing their bladder control.^19^

Silva et al.^20^ have revealed that PES can effectively reduce USDS and LUTS scores, indicating that PES can improve the quality of life for patients. The findings of this study show that the post-treatment USDS and LUTS scores in the study group were significantly lower than those in the control group, with statistically significant differences (p=0.00). Meanwhile, the post-treatment QOL score in the study group was significantly lower than that in the control group, with the post-treatment SF-36 score being significantly higher than that in the control group, both with statistically significant differences (p=0.00). This indicates that PES can effectively improve voiding symptoms and enhance quality of life, which is consistent with the findings of Silva et al. This is also due to the dual therapeutic and diagnostic effects of PES, which can deliver different intensities of bioelectrical stimulation, stimulate the pelvic floor muscles and nerves, enhance the strength and elasticity of the pelvic floor muscles, delay muscle atrophy, and instruct patients in pelvic floor muscle training based on biofeedback signals, which makes it possible for patients to control pelvic floor muscle contraction and relaxation more quickly and accurately and improve bladder function, thereby reducing voiding symptoms and enhancing their quality of life.^20^ This manuscript conform the Enhancing the Quality and Transparency Of health Research (EQUATOR) network guidelines.

### Limitations:

It included a small sample size, without grouping studies based on different causes of neurogenic bladder (NB). With more in-depth research to be conducted in the future, the sample size should be increased, follow-up content should be added, and the study design should be refined, so as to provide a more objective evaluation of this therapy while seeking more effective therapies for patients.

## CONCLUSIONS

Bladder function training combined with pelvic floor biofeedback electrical stimulation can improve the therapeutic effect, alleviate dysuria and urinary retention symptoms, facilitate patient recovery, relieve lower urinary tract symptoms, and enhance patients’ quality of life, which is worthy of clinical application.

### Authors’ Contributions:

**JG** carried out the studies, participated in collecting data, and drafted the manuscript, and are responsible and accountable for the accuracy or integrity of the work

**JZ:** Literature search, performed the statistical analysis and participated in its design. Critical Review.

All authors read and approved the final manuscript.
